# Cryoablation reshapes the immune microenvironment in the distal tumor and enhances the anti-tumor immunity

**DOI:** 10.3389/fimmu.2022.930461

**Published:** 2022-08-31

**Authors:** Ying Wu, Fei Cao, Danyang Zhou, Shuanggang Chen, Han Qi, Tao Huang, Hongtong Tan, Lujun Shen, Weijun Fan

**Affiliations:** ^1^ Department of Minimally Invasive Interventional Therapy, Sun Yat-sen University Cancer Center, Guangzhou, China; ^2^ State Key Laboratory of Oncology in South China, Collaborative Innovation Center of Cancer Medicine, Sun Yat-sen University, Guangzhou, China; ^3^ Department of Interventional Therapy, Shenzhen Second People’s Hospital, The First Affiliated Hospital of Shenzhen University, Shenzhen, China; ^4^ Department of Oncology, Peking University Shenzhen Hospital, Shenzhen, China; ^5^ Department of Oncology, Yuebei People’s Hospital, Shantou University Medical College, Shaoguan, China

**Keywords:** cryoablation, tumor microenvironment, lysosome, SNAP23, STXBP2

## Abstract

As one of the local treatments, cryoablation plays an increasingly important role in the comprehensive treatment of malignant tumors with its advantages of less trauma, high reproducibility, and minimally invasive. Activation of anti-tumor immunity, another characteristic of cryoablation, has attracted more and more attention with the extensive application of immunotherapy. Unfortunately, the mechanism by which cryoablation enhances anti-tumor immunity is still unclear. In this study, we applied a multi-omics approach to investigate the effects of local cryoablation in the distal tumor microenvironment. The results revealed that large amounts of tumor antigens were released post-cryoablation, leading to a sterile inflammatory response in distant tumors. During this period, activated lysosome-related pathways result in over-expression of SNAP23 (Synaptosome associated protein 23) and STXBP2 (Syntaxin binding protein 2), activation of immune effector cells, suppression of the release of immunosuppressive factors, and finally enhancement of anti-tumor immunity, which shows a broad prospect in combined immunotherapy.

## Introduction

Cryoablation has a long history and was first used in the 1840s to treat breast and uterine cancers (1). Cryoablation is a local treatment method with minimally invasive and practical, with fewer complications, short hospital stays, and repeatable operations (2). With the development of cryoablation technology, cryoablation has been used to treat various solid tumors, including skin, liver, lung, breast, and bone tumors (3).

With the wide application of cryoablation in clinical practice, its minimally invasive nature has been recognized, and the immune response post cryoablation has attracted more and more attention. Tumor cells are damaged by repeated freeze-thaw cycles, accompanied by the release of cell contents. Therefore, these released cell contents retain their original properties, leading to another anti-tumor mechanism after cryoablation: stimulated immunological targeting of tumor cells (1, 3–8). Previous studies have reported spontaneous resolution of distant metastases during cryoablation of prostate cancer (9, 10). Besides, other studies have shown that cryoablation reduces tumor metastasis and that the T lymphocyte in tumor-draining lymph nodes (TDLN) secrete higher levels of interferon-gamma (IFN-γ) than surgery (5). Moreover, tumors that remain *in situ* after cryoablation could protect against subsequent tumor rechallenge (11, 12).

Unfortunately, the mechanism by which cryoablation enhances anti-tumor immunity is still unclear. Previous studies reported that cell contents released by cryoablation contain “danger signals” such as pro-inflammatory cytokine, heat shock protein, DNA, and RNA recognized by toll-like receptors, which activate the natural immune response (1, 3, 4, 7, 13–15). With the wide application of the new generation of immunotherapy represented by immune-checkpoint inhibitors, cryoablation combined with immunotherapy has shown more and more broad prospects, and several clinical trials are currently exploring combination therapies (16–22). However, the timing and sequence of cryoablation, the extent of cryoablation, and the choice of target tumor are still unclear, which requires further understanding of the mechanism of cryoablation enhancing anti-tumor immunity.

In this study, we applied a multi-omics approach to investigate the effects of local cryoablation on the distal tumor microenvironment, providing a theoretical basis for cryoablation in combination with other immunotherapy regimens.

## Methods

### Cell culture and primary mouse

Mouse colon cancer MC38 was purchased from Guangzhou Saifei Trading Co., Ltd. (Jennie biological technology). Cells were regularly tested for Mycoplasma contamination by quantitative PCR. All of these cells were cultured in Dulbecco’s modified Eagle’s medium (DMEM; Gibco) supplemented with 10% fetal bovine serum (FBS; Gibco); and 100 μg/ml Penicillin/Streptomycin (Gibco).

### Primary mouse and mouse models

C57BL/6J mice were purchased from Guangdong medical laboratory animal center. Mouse xenografts were generated in 6-8-week-old C57BL/6J mice by subcutaneous implantation of 0.5×10^6^ cells (both sides). Animals were randomized into two groups (control VS cryoablation) for collection and analysis. On the 7th day, the subcutaneous tumor of the mice grew to about 5-6 mm in diameter. After anesthesia, one side of the tumor was subjected to cryoablation. The cryoablation was performed using Visual-ICE™ System (Galil Medical, Israel). The cryoprobe was inserted into the targeted lesions, and two 40-sec freezing cycles, separated by a 20-sec freezing and an active 20-sec thawing session, were performed. The subcutaneous tumor achieved complete ablation. On the contralateral tumor, two orthogonal diameters were measured every 2-3 days (control vs. cryoablation). Tumor volume was calculated as V = π/6 × L × W × H, where L, W, and H represent the length, width, and height, respectively. All experiments with mice were performed by protocols approved by the Sun Yat-sen University Cancer Center (SYSUCC). The experiments were repeated at least two times.

### Immune profiling by flow cytometry

Implants of the indicated MC38 cell populations in D1, D3, D5, D7, D9 and were measured for total weight. Biopsies were then minced using scalpels and digested with 500 U/ml Collagenase IV (Sigma–Aldrich) and 0.02 mg/ml DNAse I (Coolaber) and 300 mg/ml-Hyaluronidase (Solarbio) per 0.3 g tumor weight for 30-50 min at 37°C. After digestion, the cell suspension was passed through a 40 μm cell strainer to remove large pieces of undigested tissue. Erythrocytes were lysed using Red Blood Cell Lysis Solution (TIANGEN; RT122-02).

In terms of cell surface stain, 1×10^6^ cells were incubated with an anti-Fc receptor blocking antibody and stained with the indicated antibodies in stain buffer (BD) for 30 min on ice. Viability was assessed by staining with Fixable Viability Stain 620 (BD). For intracellular staining of Foxp3, cells were first fixed and permeabilized using a Fixation/Permeabilization Solution Kit (BD). All flow cytometry was performed on a CytoFLEX LX (Beckman Coulter). The flow cytometry data was analyzed using FlowJo V10.6.2 (BD). Flow cytometry antibodies were used as follows (all from BD): Anti-CD45 FITC (Clone 30-f11), Anti- CD8a APC-H7 (Clone 53-6.7), Anti- CD4 BV510 (Clone RM4-5), Anti- CD279 BB700 (Clone J43), Anti- IFN-Gma PE-Cy7 (Clone XMG 1.2), Anti- Foxp3 Alexa 647 (Clone MF23), Anti- LY-6G BV786 (Clone 1A8), Anti- CD11b BV605 (Clone M1/70), Anti- CD16/CD32 Pure (Clone 2.4G2). The experiments were repeated at least two times.

After gating single cell types and excluding non-viable cells by staining with Fixable Viability Stain 620 and excluding debris with side scatter area and forward scatter area, the cells are identified using the following combination of cell markers: CD4^+^ T cell: CD45^+^CD4^+^; CD8^+^ T cell: CD45^+^CD8^+^; Treg cell: CD45^+^CD4^+^Foxp3^+^.

### Bulk RNA-seq and data analysis

Extract mRNA with a magnetic bead adsorption method and cut it into a small fragment to synthesize cDNA. The RNA samples were sequenced by the Shanghai Personalbio Company. The library preparations were sequenced on a NovaSeq platform after cluster generation. Differential expression analysis was performed using the edgeR R package (3.18.1). The P values were adjusted using the Benjamini & Hochberg method, and the absolute foldchange of 1 and P-value of 0.05 were set as the threshold for identification of differentially expressed genes. STRING database was used for Protein-protein interaction network analysis (PPI) analysis. CIBERSORT was used for calculating immune cell infiltration in the tumor.

### Quantitative proteomics and data analysis

Pre- (3 samples) and post-cryoablation (4 samples) samples were obtained from advanced melanoma patients who had progressed after a series of therapies, including chemotherapy, targeted therapy, and immunotherapy. We obtained tumor tissue from the same distal tumor (without cryoablation) by fine-needle aspiration biopsy before and three weeks after cryoablation. The patients and Ethics Committee have approved all procedures. No related complications occurred in all patients. The samples were frozen in liquid nitrogen and ground with a pestle and mortar. The protein samples were sequenced in Shanghai Genechem Co., LTD (4D Label-free). The MS data were analyzed using MaxQuant software version 1.6.14.0. Samples were analyzed on a nanoElute (Bruker, Bremen, Germany) coupled to a timsTOF Pro (Bruker, Bremen, Germany) equipped with a CaptiveSpray source, and the timsTOF Pro (Bruker, Bremen, Germany) was operated in PASEF mode. The global false discovery rate (FDR) cutoff for peptide and protein identification was set to 0.01. Proteins which Fold change>2 or <0.5 and p-value (Student’s t-test) <0.05 were considered to be differentially expressed proteins. We applied the SangerBox tool to further bioinformatics analysis of DEGs (sangerbox.com).

### Statistical Analysis

For categorical variables versus categorical variables, Fisher’s exact test was used; for categorical variables versus continuous variables, the Kruskal-Wallis test was used to test if any of the differences between the subgroups were statistically significant. Tumor growth curves were analyzed by one- and two-way analysis of variance (ANOVA). All statistical tests were two-sided, and statistical significance was considered when p value <0.05 unless otherwise indicated. All the analyses of clinical data were performed in R (version 4.1.1). R software, with limma, ggplot2, dplyr, reshape2, RColorBrewer packages, etc.

## Result

### Cryoablation could prolong the survival of mice without a significant effect on the distant tumor volume control

**Figure 1:**
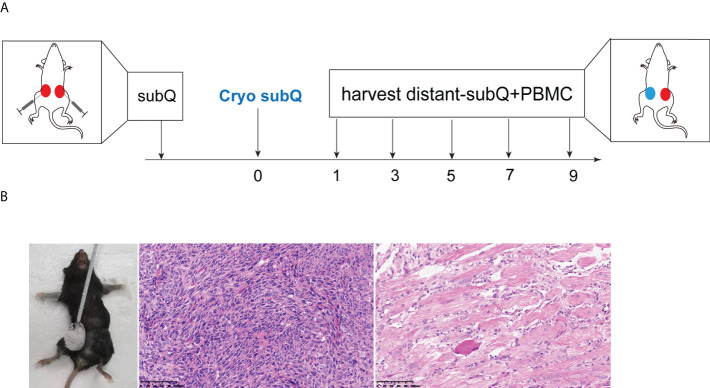
Animal model of cryoablation *in vivo*. **(A)** Mouse xenografts were generated in 6-8-week-old C57BL/6J mice by subcutaneous implantation of 0.5×10^6^ MC38 cells (both sides). On the 7th day, one side of the tumor was subjected to cryoablation; **(B)** The histopathology examination at D14 days after cryoablation showed complete ablation of the subcutaneous tumor (20x).

On the D7 day, the subcutaneous tumor of the mice grew to about 5-6mm in diameter. After anesthesia, one side of the tumor was subjected to cryoablation (**Figure 1A**). The cryoprobes were inserted into the targeted lesions, and two 40-sec freezing cycles, separated by a 20-sec freezing and an active 20-sec thawing session, were performed. The histopathology examination at D14 day after cryoablation showed complete ablation of the subcutaneous tumor (**Figure 1B**). Post-cryoablation, the tumor appeared to have coagulative necrosis with mang inflammatory cell infiltration.

**Figure 2:**
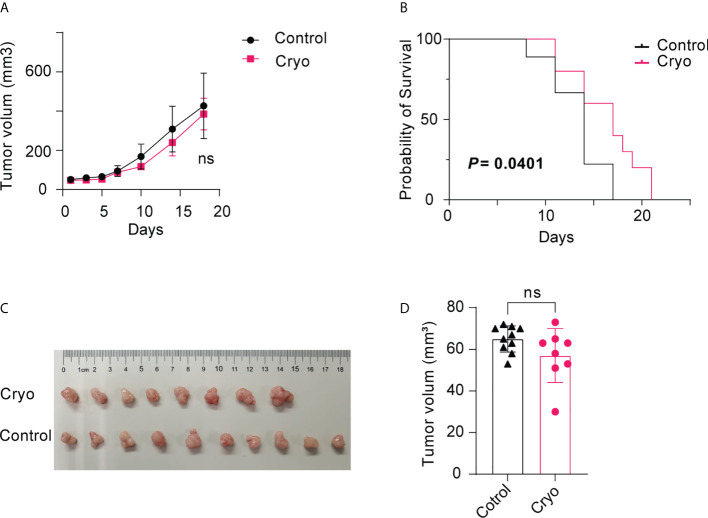
**(A)** There was no significant difference in tumor volume between the cryoablation (5 mice) and the control group (5 mice); **(B)** The survival time of mice in the cryoablation group was significantly prolonged than control group post cryoablation; **(C,D)** There was no significant difference in tumor volume between the control (10 mice) and the cryoablation (8 mice) group on the D5 day after cryoablation. The experiments were repeated at least two times. ns, not statistically significant.

Two orthogonal diameters were measured on the contralateral tumor every 2-3 days. There was no significant difference in tumor volume between the cryoablation group and the control group (**Figures 2A–D**). However, the survival time of mice in the cryoablation group was significantly prolonged post cryoablation (**Figure 2B**).

### Cryoablation increased the number and infiltration of immune effector cells in distant tumors

**Figure 3:**
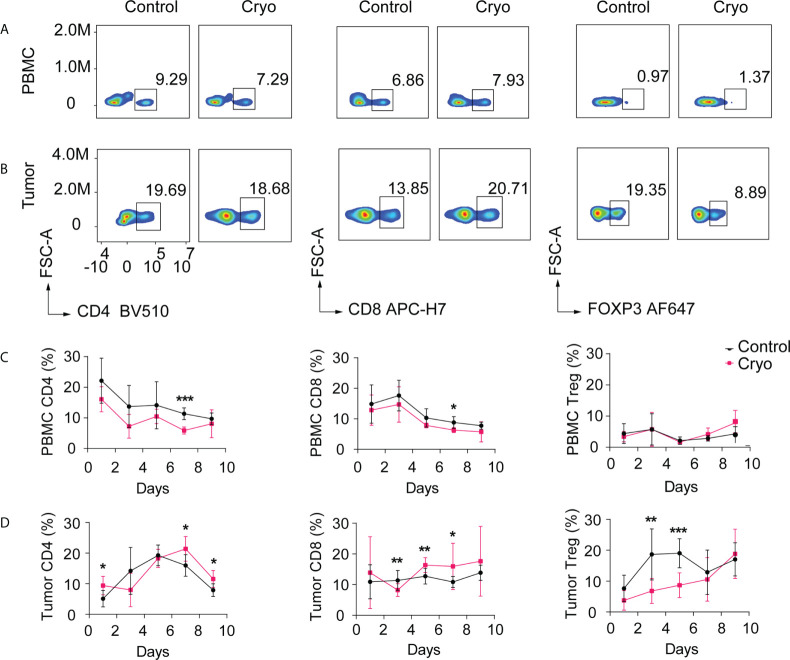
**(A, B)** Flow chart of CD4^+^ T cells, CD8^+^ T cells, Treg cells in PBMC, and distal tumor on day 5. The distant tumors and peripheral blood mononuclear cells (PBMC) of mice were detected by flow cytometry on days D1, D3, D5, D7, and D9 post cryoablation. **(C)** Changes in the proportion of CD4^+^ T cells, CD8^+^ T cells, and Treg cells in peripheral blood (PBMC); **(D)** Changes in the proportion of CD4^+^ T cells, CD8^+^ T cells, and Treg cells in the distant tumor (non-cryoablation tumors). *: p<0.05, **: p<0.01, ***: p<0.001, ns: not statistically significant. The experiments were repeated at least two times.

To further study the changes in immune microenvironment in distant tumors of mice, the distant tumors and peripheral blood mononuclear cell (PBMC) of mice were detected by flow cytometry on days D1, D3, D5, D7, and D9 post cryoablation. The results indicated that the changes in immune cells, including CD4, CD8, and Treg cells in the PBMC of mice, were not significant post cryoablation (**Figures 3A, C**).

However, the immune cells isolated from the distant tumor post cryoablation varied significantly over time. In distant tumors, CD4^+^ T cells in the cryoablation group were significantly higher than those in the control group on the first-day post cryoablation. There was no difference between the two groups on D3-D5 days, but there was a significant increase after D5 days in the cryoablation group. CD8^+^ T cells increased significantly on the D5-D7 days post cryoablation, but there was no significant difference between the two groups on the D9 day. Treg cells decreased substantially on the D3-D6 days post cryoablation, but there was no significant difference between the two groups on the D7 day (**Figures 3B, D**).

The immunohistochemical results showed that most of the immune cells in the distant tumor were located in the periphery of the tumor, and few could infiltrate into the tumor (**Figure S1**). The results of immunohistochemical were primarily consistent with those of flow cytometry. CD8^+^ T cells in the cryoablation group were more than those in the control group on D5-D7 days and infiltrated more into the tumor.

### Bulk RNA-seq results indicated that cryoablation could reverse the immunosuppressive environment of distal tumors and enhance the anti-tumor immune

To further study the molecular mechanism of the enhancement of anti-tumor immunity by cryoablation, the mice were sacrificed on the 5th day after cryoablation. Then the contralateral tumors (non-cryoablation tumors) were frozen in liquid nitrogen for 15 min and stored at -80°C, parallel bulk RNA sequencing. D5 day was chosen because there were more CD8^+^ T cells in the distal tumors and fewer immunosuppressive Treg cells.

**Figure 4:**
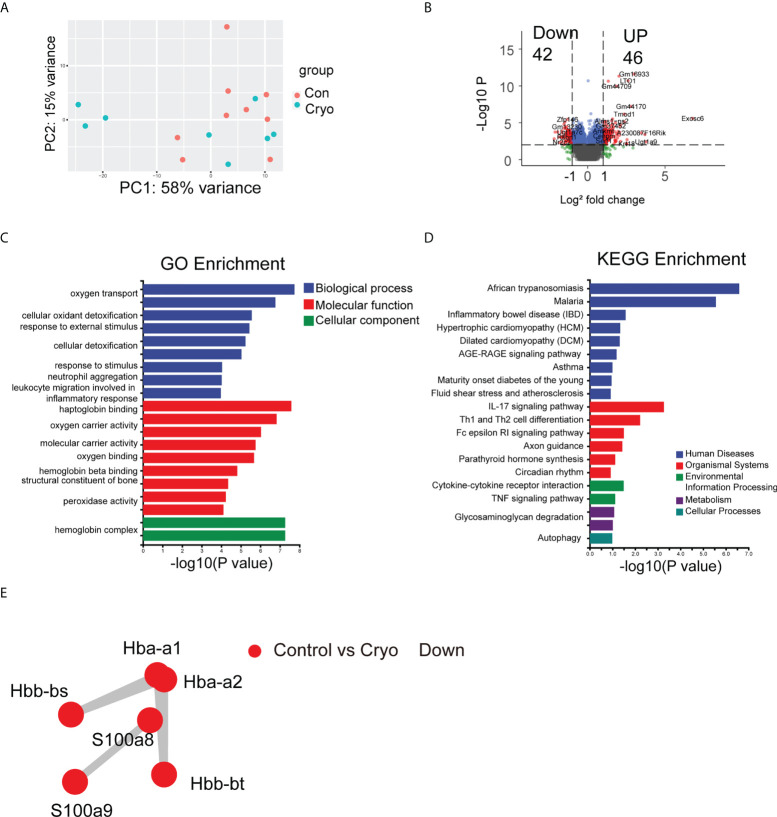
The results of bulk RNA-seq. **(A)** PCA analysis of the RNA-seq in the cryoablation group (9 samples) and the control group (8 samples) showed no significant clustering between the two groups; **(B)** The Volcano plot revealed that 42 genes were up-regulated and 42 genes were down-regulated (Control vs Cryo); **(C,D)** GO and KEGG enrichment analysis results; **(E)** The PPI results showed that the major interacting DEGs were Hba-a1, Hba-a2, Hbb-bs, Hbb-bt, S100A8, and S100A9 (Control vs Cryo down-regulated).

Through linear transformation, Principal Components Analysis (PCA) reduces high-dimensional data to two-dimensional or three-dimensional while maintaining the characteristics of the most significant contribution of variance, reducing data complexity. PCA analysis of the RNA-seq in the cryoablation group (9 samples) and the control group (8 samples) showed no significant clustering between the two groups (**Figure 4A**). The differentially expressed genes were analyzed by DESeq, and the conditions of the differentially expressed genes were: multiple of expression |log2foldchange| > 1, significant p-value < 0.05 (**Table S1**). The results revealed that 46 genes were up-regulated, and 42 were down-regulated (Control vs. Cryo) (**Figure 4B**).

We used topGO for GO enrichment analysis to determine the GO term in which the differentially expressed genes (DEGs) were significantly enriched compared with the whole genome background and to determine the main biological functions of the DEGs (**Table S2**). Among the DEGs, we found a significant enrichment of biological processes (BP) about the “Oxygen transport,” “Response to external stimulus,” “Response to stimulus,” and the “Neutrophil aggregation”; Cellular component (CC) pertaining to the “Hemoglobin complex related” and the “Extracellular related”; Molecular function pertaining to the “Haptoglobin binding” (**Figure 4C**). Besides, we use ClusterProfiler for KEGG enrichment analysis (**Table S3**). Some of the pathways are related to the organismal systems, environmental information processing, and cellular processes, including “IL-17 signaling pathway”, “Th1 and Th2 cell differentiation”, “Cytokine-cytokine receptor interaction,” “TNF signaling pathway,” and “Autophagy” so on (**Figure 4D**).

Protein-protein interaction network analysis (PPI) is an analysis that reveals the interaction between genes. Analyze the prediction of the interaction relationship of DEGs using the STRING database. The results showed that the significant interacting DEGs were Hba-a1, Hba-a2, Hbb-bs, Hbb-bt, S100A8, and S100A9 (Control vs. Cryo down-regulated) (**Figure 4E**).

### Cryoablation enhanced anti-tumor effect through the lysosome-related pathway

To further analyze the effect of cryoablation on the microenvironment of distant tumors at the protein level, we collected tumor samples from patients with advanced melanoma pre- (3 samples) and post-cryoablation (4 samples).

**Figure 5:**
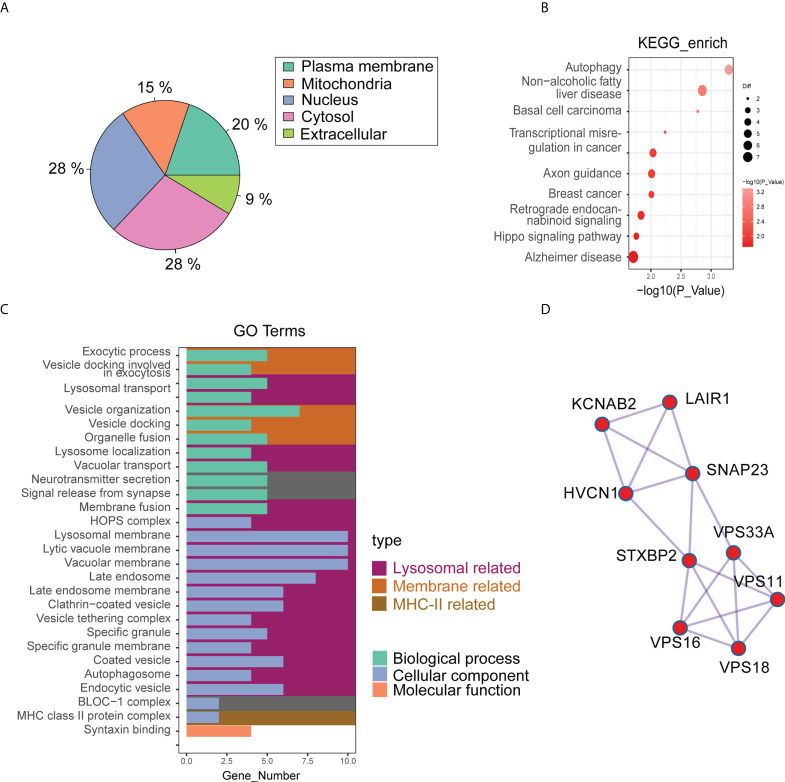
The results of quantitative proteomics (4D Label-free) of pre- (3 samples) and post-cryoablation (4 samples). **(A)** The differential proteins’ subcellular localization; **(B,C)** GO and KEGG enrichment analysis results; **(D)** The PPI results showed that the major interacting differentially expressed proteins were KCNAB2, LAIR1, HVCN1, SNAP23, STXBP2, VPS33A, VPS11, VPS16, and VPS18.

The cluster analysis results showed significant differences in protein grouping between pre-and post-cryoablation (**Figure S2B**). The results of differential proteins showed that there were 31 up-regulated proteins and 50 down-regulated proteins (pre- vs. post-cryoablation) (**Figure S2A**) (**Table S4**). Further analysis of differential proteins’ subcellular localization revealed that 28% were located in the nucleus, 28% in the cytosol, 20% in the plasma membrane, 15% in the mitochondria, and 9% in the extracellular (**Figure 5A**). The analysis of the domain of differential proteins showed that it was mainly related to “Sorbin and SH3 domain-containing protein,” “SoHo domain,” “C3HC4 Zinc finger,” and “Pleckstrin homology domain,” “MHC class II-beta chain-N terminal” and so on (**Figure S2C**). However, there is no overlap between the differentially expressed proteins of the protein spectrum and the DEGs of the bulk RNA-seq (**Figure S2D**).

**Figure 6:**
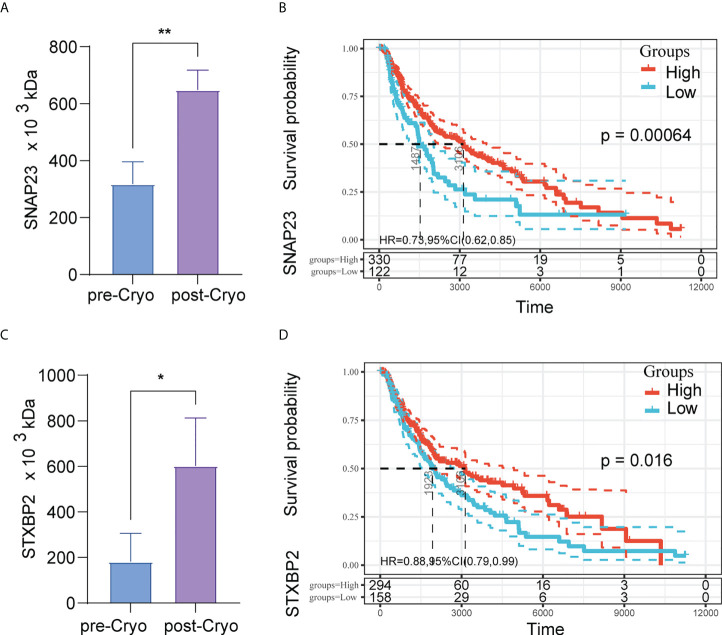
The expression of SNAP23 and STXBP2 proteins increased significantly in distant tumors post cryoablation **(A,C)**; High expression of protein SNAP23 (HR: 0.73, 95%CI:0.62-0.85, P=0.00064) **(B)** and STXBP2 (HR: 0.88, 95%CI:0.79-0.99, P=0.016) **(D)** are associated with better prognosis in TCGA-SKCM (N=452).

Among the differential proteins, we found a significant GO enrichment of pathways pertaining to the “lysosomal related,” “membrane related,” and “MHC-II related” (**Figure 5C**) (**Table S5**). Besides, we also found a significant KEGG enrichment of pathways pertaining to autophagy, non-alcoholic fatty liver disease, basal cell carcinoma, Hippo signaling pathway, and so on (**Figure 5B**) (**Table S6**). PPI analysis used the STRING database to predict interactions between differential proteins. The results showed that the significant interacting differentially expressed proteins were KCNAB2, LAIR1, HVCN1, SNAP23, STXBP2, VPS33A, VPS11, VPS16, and VPS18 (**Figure 5D**). Among these proteins, proteins associated with the lysosomal pathway found in GO enrichment analysis were SNAP23, STXBP2, VPS33A, VPS11, and VPS18. Moreover, the expression of SNAP23 and STXBP2 proteins increased significantly in distant tumors post cryoablation (**Figures 6A, C**).

Further to investigate the relationship between SNAP23 and STXBP2 and patient survival, we further analyzed the pan-cancer data in TCGA. The results showed that high expression of SNAP23 was a protective factor in SKCM (Skin Cutaneous Melanoma) and KIRC (Kidney renal clear cell carcinoma). In contrast, high expression of STXBP2 was a protective factor in BLCA (Bladder Urothelial Carcinoma), SARC (Sarcoma), SKCM, THYM (Thymoma), and CHOL (Cholangiocarcinoma) (**Figure S3**). High expression of protein SNAP23 (HR: 0.73, 95%CI:0.62-0.85, P=0.00064) (**Figure 6B**) and STXBP2 (HR: 0.88, 95%CI:0.79-0.99, P=0.016) (**Figure 6D**) are associated with better prognosis in SKCM.

**Figure 7:**
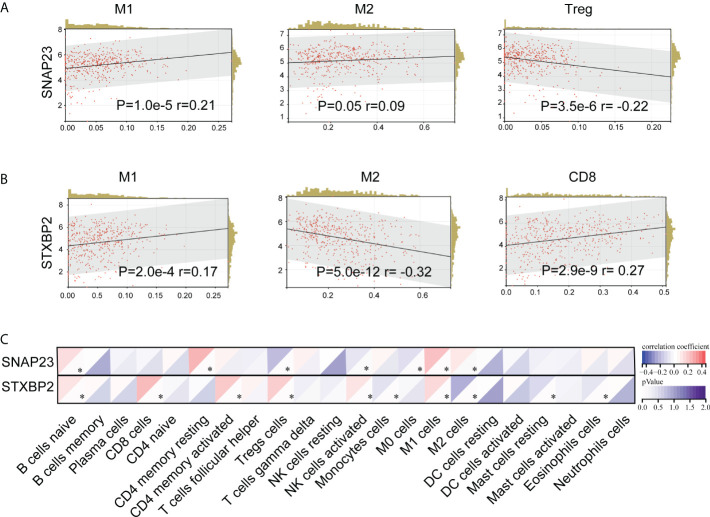
CIBERSORT analysis of the effect of SNAP23 **(A)** and STXBP2 **(B)** genes on TCGA-SKCM (N=452) tumor immune microenvironment **(C)**..

Moreover, tumor tissue does not simply contain tumor cells. It comprises various types of cells, including stromal cells, fibroblasts cells, immunocytes, etc., which constitute the tumor microenvironment (TME). CIBERSORT is a commonly used method for calculating immune cell infiltration. We analyzed the impact of SNAP23 and STXBP2 genes on the immune micro-environment in SKCM tumors through CIBERSORT (**Figure 7C**). The results showed that the expression of SNAP23 was positively correlated with the number of M1 cells and negatively correlated with the number of Treg cells (**Figure 7A**). The expression of STXBP2 was positively correlated with the expression of M1 cells, CD8 cells, and NK-activated cells, but negatively correlated with the expression of M2 cells (**Figure 7B**).

## Discussion

As one of the local treatments, cryoablation plays an increasingly important role in the comprehensive treatment of malignant tumors with its advantages of less trauma, high reproducibility, and minimally invasive (23). With the development of cryoablation technology, cryoablation has been used to treat various solid tumors, including skin tumors, liver cancer (24), lung cancer (25), breast cancer (26), and other tumors (22, 23, 27, 28).

Since the 1970s, cryoablation has been reported to stimulate humoral immunity and induce the disappearance of metastatic tumors (29, 30). Compared with partial hepatectomy, partial liver cryoablation has been reported to cause a systemic inflammatory response associated with distant organ injury and NF-κb-dependent cytokine overproduction (31–33). Current research suggests that due to the previously shielded tumor antigens being continuously exposed to the immune system, the unique function of ablation provides a therapeutic opportunity for immune stimulation (3). However, the inner mechanism of the enhancement of anti-tumor immunity induced by cryoablation is still unclear. Current studies suggest that tumor antigens, previously blocked, are continuously exposed to the immune system post cryoablation, providing therapeutic opportunities for immune stimulation.

In this study, we applied a multi-omics approach to investigate the effects of local cryoablation on the distal tumor microenvironment. *In vivo*, we demonstrated that cryoablation altered the immune microenvironment of distant tumors, resulting in an increase in immune effector cells and a decrease in immunosuppressive Treg cells, M2 macrophages cells, and thus prolonged mouse survival. A large amount of cell debris and contents were released post cryoablation, so we analyzed the proteomics of the samples pre- and post-cryoablation. The results revealed that the lysosome- and MHC-II-related pathways were activated after cryopreservation, and the expression of SNAP23 and STXBP2 increased significantly after cryopreservation. Further analysis of TCGA pan-cancer data revealed that both SNAP23 and STXBP2 overexpression were associated with a better prognosis of SKCM. Moreover, the analysis of the tumor microenvironment of SKCM in TCGA showed that the expression of SNAP23 was positively correlated with the number of immunes enhancing M1 cells while negatively correlated with the number of immunosuppressive immune Treg cells. The expression of STXBP2 was positively correlated with the number of immune-enhancing M1 and CD8 cells while negatively correlated with the number of immunosuppressive immune M2 cells.

Gazzaniga, S et al. found that extensive edema and proliferation of fibroblasts first appeared in the tumor ablation region post-cryoablation, followed by collagen accumulation. During this period, a large number of neutrophil cells began to accumulate around the tumor post-cryoablation at 1-3 days, and a large number of macrophages at 3-7 days, and then both gradually decreased (34). The reaction in and around the tumor was intense for about seven days and then gradually reduced to about two weeks. During this period, many tumors interact with immune cells and may affect the microenvironment of distant tumors (34). In this study, we found an increase in immune effector cells and a decrease in immunosuppressive Treg cells in distant tumors post-cryoablation. Since the effect did not last long and only about a week, it did not control the tumor volume immediately, but it significantly prolonged the survival of the mice. In the analysis of the immune microenvironment of Bulk RNA-seq, it was also found that cryoablation could change the immune microenvironment of distant tumors, increase effector cells and decrease immunosuppressive cells. Activation of response to stimulus, neutrophil aggregation, IL-17 signaling, and TNF signaling pathway post-cryoablation may be the reason for changes in the distal tumors’ immune microenvironment.

S100A8/A9 protein belongs to the Ca2 + combined with the S100 protein family (35). S100A8/A9 is mainly secreted from activated neutrophils and monocytes. Moreover, S100A8/A9 is important in regulating the inflammatory process by stimulating leukocyte aggregation and inducing cytokine secretion (36–38). In this study, S100A8/A9 was significantly enriched post-cryoablation in PPI analysis. This indicated that cryoablation promotes inflammation in distant tumors, leading to enhanced anti-tumor immunity.

Cryoablation destroys the tumor cell membrane at a low temperature and releases a large number of cell structures and cell contents, which can well maintain the original structure of cell contents and expose to a large number of effective tumor antigens (39). Previous studies reported that cell contents released by cryoablation contain “danger signals” such as pro-inflammatory cytokine, heat shock protein, DNA, and RNA recognized by toll-like receptors, which activate the natural immune response (1, 13, 40). Further analysis of the changes in protein in distant tumors pre- and post-cryoablation revealed that the significant activation pathways were the lysosomal-related, membrane-related, and MHC-II-related pathways. Therefore, it is demonstrated that cryoablation could induce the release of tumor-associated antigens and activate the anti-tumor immune function of distal tumors.

Synaptosome associated protein 23 (SNAP23) is an essential component of membrane insertion machinery (41). Its related pathways are “Class I MHC mediated antigen processing and presentation” and “Vesicle-mediated transport.” SNAP23 and its partner SNAREs mediate the fusion of the plasma membrane with intracellular organelles or vesicles to form phagosomes, as well as the fusion of phagosomes with endosomes or lysosomes to induce phagosome maturation, characterized by reactive oxygen species production and acidification (42). Syntaxin binding protein 2 (STXBP2) is involved in intracellular trafficking, control of SNARE (soluble NSF attachment protein receptor) complex assembly, and the release of cytotoxic granules by natural killer cells (43–45). In this study, we found that SNPA23 and STXBP2 expression increased post-cryoablation significantly.

Further analysis of TCGA data showed that both SNAP23 and STXBP2 were positively correlated with the number of SKCM immune effector cells but negatively correlated with the number of immunosuppressive cells. Furthermore, high expression of SNAP23 and STXBP2 was associated with a better prognosis of SKCM. Notch1 (Notch Receptor 1) is critical in modulating melanoma tumor cell growth and survival (46). Besides, Notch1 inhibition improves tumor responses to immune checkpoint inhibitors. Notch1 contributes to an immune-suppressive tumor microenvironment by inhibiting the expression of SNAP23 and overexpression of IL-6, IL-8, and CCL5 downstream of the pathway, thus affecting the efficacy of immune-checkpoint inhibitors (47). This provides a possible internal mechanism for the expression of SNAP23 to increase post-cryoablation, change the immune microenvironment in distant tumors, and improve prognosis.

In summary, this study applied a multi-omics approach to investigate the effects of local cryoablation on the distal tumor microenvironment. Those results proved that large amounts of tumor antigens are released post-cryoablation, leading to a sterile inflammatory response in distant tumors. During this period, lysosome-related pathways are activated, resulting in overexpression of SNAP23 and STXBP2, activation of immune effector cells, suppression of the release of immunosuppressive factors, and finally, enhancement of anti-tumor immunity.

Despite the great potential of immune-checkpoint inhibitors, many cancer patients still do not respond well to treatment (The efficacy of the single drug is only 20-40%), mainly due to the lack of tumor antigens, lymphocytes, and immunosuppressive tumor microenvironment (48). Combined with immune-checkpoint inhibitors, cryoablation can release many original, undenatured tumor-associated antigens, producing a more potent anti-tumor immune effect (3, 8, 49). In our previous studies, it has been demonstrated that cryoablation combined with Pembrolizumab (CATAP) could significantly improve the objective response rate in patients with liver metastases from melanoma with minimal side effects (22). Even one patient with diffuse liver metastasis achieved complete remission (CR). At the same time, cryoablation combined with the immune adjuvant, cell therapy, immune-checkpoint inhibitors, and other treatment methods is also being explored.

## Data availability statement

The data presented in the study are deposited in the NCBI, GEO DataSets repository, accession number GSE201590.

## Ethics statement

The studies involving human participants were reviewed and approved by Sun Yat-sen University Cancer Center (SYSUCC). The patients/participants provided their written informed consent to participate in this study. The animal study was reviewed and approved by Sun Yat-sen University Cancer Center (SYSUCC).

## Author contributions

YW, CF, TH, DZ, HQ, and HT performed experiments. WF and LS planned the study and analyzed the data. YW and SC wrote the manuscript. All authors contributed to, read, and approved the final manuscript.

## Funding

This work was funded by the Guangdong Provincial Key R&D Programme. Grant number: 2019B110233001.

## Conflict of interest

The authors declare that the research was conducted in the absence of any commercial or financial relationships that could be construed as a potential conflict of interest.

## Publisher’s note

All claims expressed in this article are solely those of the authors and do not necessarily represent those of their affiliated organizations, or those of the publisher, the editors and the reviewers. Any product that may be evaluated in this article, or claim that may be made by its manufacturer, is not guaranteed or endorsed by the publisher.

## Supplementary material

Supplementary Figure 1 The immunohistochemical results showed that most of the immune cells in the distant tumor were located in the periphery of the tumor and few could infiltrate into the tumor. Red arrow: CD8+ T cells in the cryoablation group were more than those in the control group on D5-D7 days and infiltrated more into the tumor.

Supplementary Figure 2
**(A)** The results of differential proteins showed that there were 31 up-regulated proteins and 50 down-regulated proteins (pre- vs. post-cryoablation); **(B)** The cluster analysis results showed significant differences in protein grouping between pre-and post-cryoablation; **(C)** The analysis results of the domain of differential proteins; **(D)** There is no overlap between the differentially expressed proteins of the protein spectrum and the DEGs of the bulk RNA-seq.
