# Supplementary figures and images for: Cryoablation reshapes the immune microenvironment in the distal tumor and enhances the anti-tumor immunity
Source: Front Immunol. 2022 Aug 31;13:930461. doi: 10.3389/fimmu.2022.930461 (PMC9470839; doi:10.3389/fimmu.2022.930461)

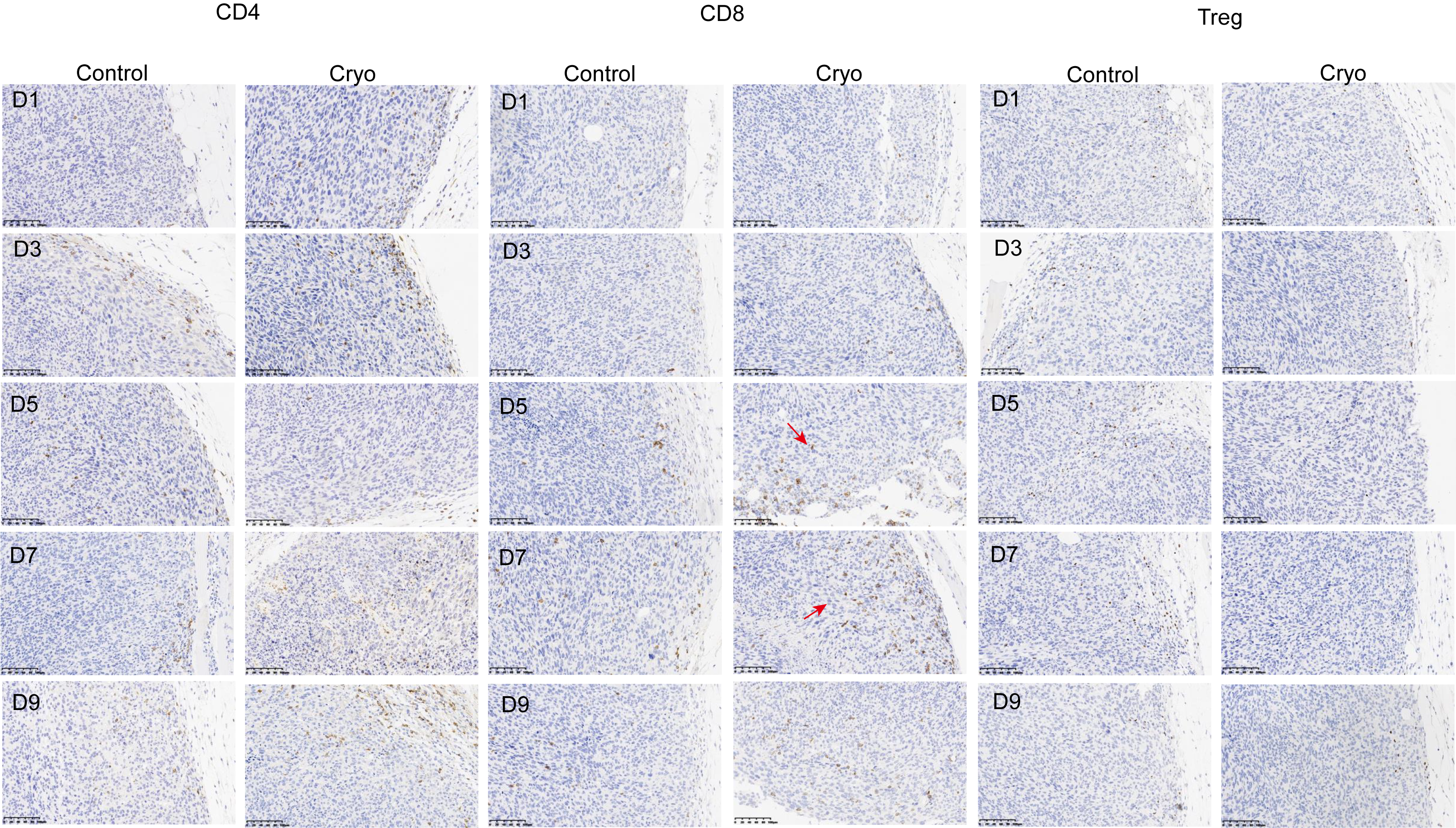

Supplement: Supplementary Figure 1 — The immunohistochemical results showed that most of the immune cells in the distant tumor were located in the periphery of the tumor and few could infiltrate into the tumor. Red arrow: CD8+ T cells in the cryoablation group were more than those in the control group on D5-D7 days and infiltrated more into the tumor. [file Image_1.tif]

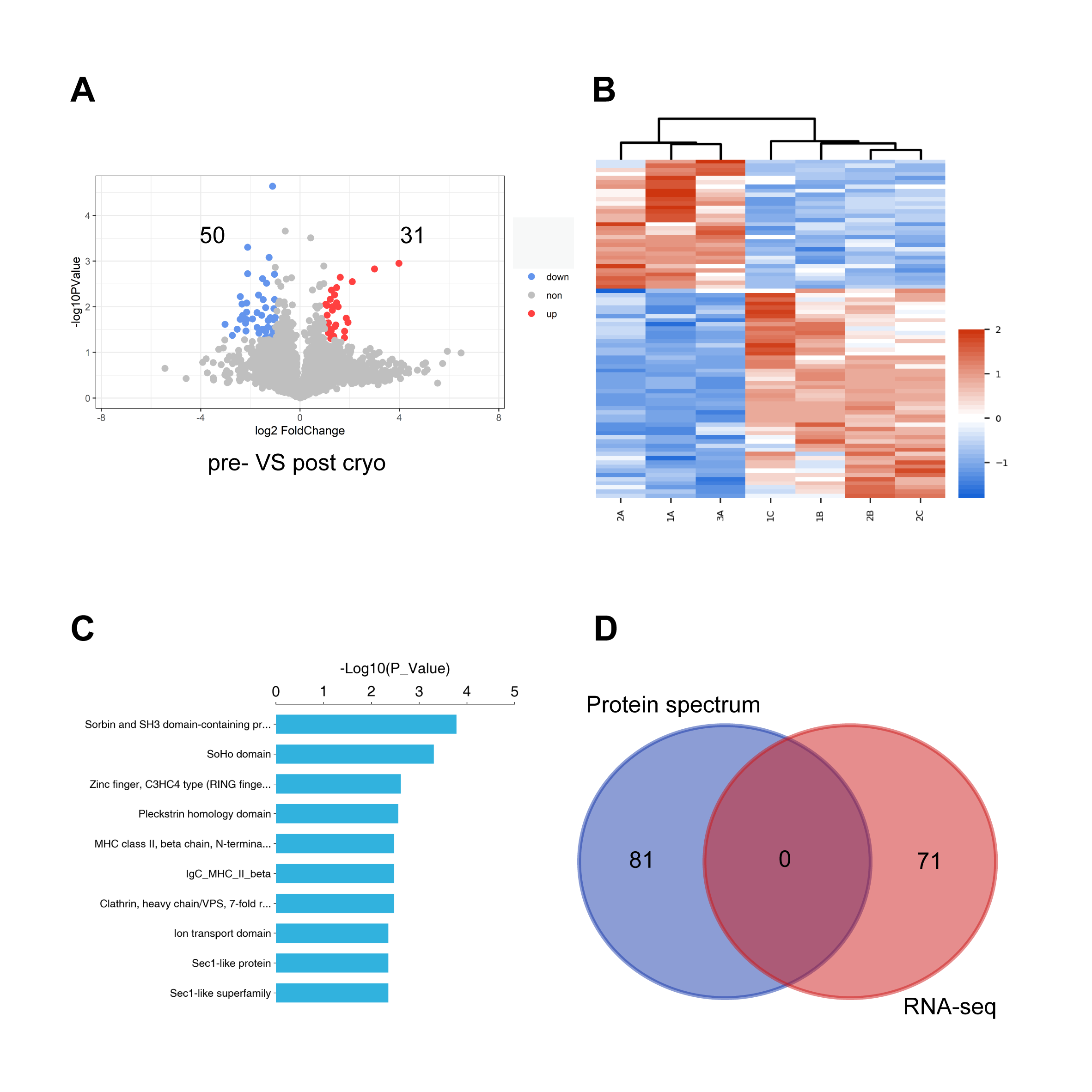

Supplement: Supplementary Figure 2 — (A) The results of differential proteins showed that there were 31 up-regulated proteins and 50 down-regulated proteins (pre- vs. post-cryoablation); (B) The cluster analysis results showed significant differences in protein grouping between pre-and post-cryoablation; (C) The analysis results of the domain of differential proteins; (D) There is no overlap between the differentially expressed proteins of the protein spectrum and the DEGs of the bulk RNA-seq. [file Image_2.tif]

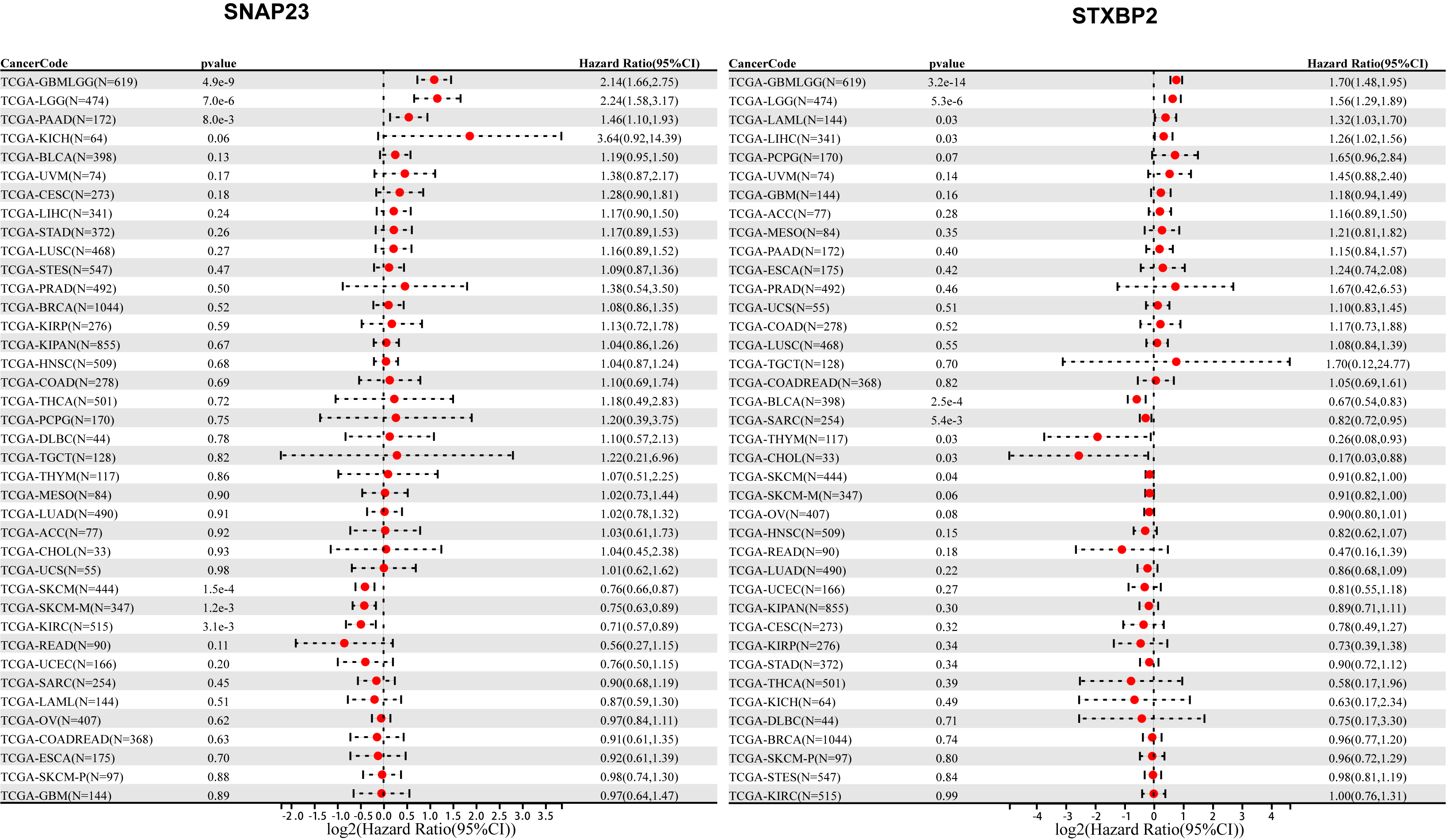

Supplement: Supplementary file 3 [file Image_3.tif]
